# Customized Chromosomal Microarrays for Neurodevelopmental Disorders

**DOI:** 10.3390/genes16080868

**Published:** 2025-07-24

**Authors:** Martina Rincic, Lukrecija Brecevic, Thomas Liehr, Kristina Gotovac Jercic, Ines Doder, Fran Borovecki

**Affiliations:** 1 School of Medicine, Croatian Institute for Brain Research, University of Zagreb, Salata 12, 10000 Zagreb, Croatia; lbr25@hotmail.com; 2 Jena University Hospital, Institute of Human Genetics, Friedrich Schiller University, Am Klinikum 1, 07747 Jena, Germany; thomas.liehr@med.uni-jena.de; 3 Department for Personalized Medicine, University Hospital Center Zagreb, 10000 Zagreb, Croatia; kristina.gotovac@kbc-zagreb.hr (K.G.J.); fran.borovecki@kbc-zagreb.hr (F.B.); 4 Pediatric Primary Health Care, Health Center Zagreb-East, 10000 Zagreb, Croatia; inesblazekovic@yahoo.com; 5 Department of Neurology, University Hospital Center Zagreb, 10000 Zagreb, Croatia; 6 Department for Functional Genomics, Center for Translational and Clinical Research, University Hospital Center Zagreb, School of Medicine, University of Zagreb, 10000 Zagreb, Croatia

**Keywords:** NDDs, ASD, CNV, custom CMA, protein-protein interaction networks, 12q24.32-q24.33, 21q22.2, 6q27, 15q13.2

## Abstract

Background: Neurodevelopmental disorders (NDDs), including autism spectrum disorder (ASD), are genetically complex and often linked to structural genomic variations such as copy number variants (CNVs). Current diagnostic strategies face challenges in interpreting the clinical significance of such variants. Methods: We developed a customized, gene-oriented chromosomal microarray (CMA) targeting 6026 genes relevant to neurodevelopment, aiming to improve diagnostic yield and candidate gene prioritization. A total of 39 patients with unexplained developmental delay, intellectual disability, and/or ASD were analyzed using this custom platform. Systems biology approaches were employed for downstream interpretation, including protein–protein interaction networks, centrality measures, and tissue-specific functional module analysis. Results: Pathogenic or likely pathogenic CNVs were identified in 31% of cases (9/29). Network analyses revealed candidate genes with key topological properties, including central “hubs” (e.g., *NPEPPS*, *PSMG1*, *DOCK8*) and regulatory “bottlenecks” (e.g., *SLC15A4*, *GLT1D1*, *TMEM132C*). Tissue- and cell-type-specific network modeling demonstrated widespread gene involvement in both prenatal and postnatal developmental modules, with glial and astrocytic networks showing notable enrichment. Several novel CNV regions with high pathogenic potential were identified and linked to neurodevelopmental phenotypes in individual patient cases. Conclusions: Customized CMA offers enhanced detection of clinically relevant CNVs and provides a framework for prioritizing novel candidate genes based on biological network integration. This approach improves diagnostic accuracy in NDDs and identifies new targets for future functional and translational studies, highlighting the importance of glial involvement and immune-related pathways in neurodevelopmental pathology.

## 1. Introduction

Disorders of the nervous system characterized by cognitive impairments and behavioral abnormalities, commonly referred to as neurodevelopmental disorders (NDDs), are of great interest to researchers and the general public. Depending on the inclusion criteria, NDDs affect 1–3% of the world’s population, with a higher prevalence in males [1]. In recent decades, remarkable genetic research efforts have identified several hundred genes associated with NDDs [2,3]. The genetic basis of NDDs, particularly autism spectrum disorders (ASD), is diverse regarding genetic research and functional genomics, ranging from syndromic genetic disorders with heritable to sporadic de novo mutations [4]. However, translating genetic research into a mechanistic understanding of NDDs and ASD will only be possible once the specific effects of genes on neurodevelopment are understood [5]. Furthermore, it is challenging to determine which genes should be prioritized for functional analysis [6]. Structural variations (including copy number variants—CNVs), single-nucleotide variants (SNVs), and insertion-deletion variants (indels) are now well-described etiological backgrounds for NDDs. Whole-genome testing is now widely available, affordable, and used to diagnose genetic disorders, but determining the pathogenicity of variants remains a challenge in diagnostic decision-making. Therefore, evidence-based knowledge from clinical practice is required when working with NDDs [7,8,9]. Here, we present data on customized chromosomal microarrays (CMA) in patients with complex NDD and/or ASD. The main objectives of the research were to use a systems biology approach based on customized CMA in selected patients with NDD and/or ASD to investigate a large number of candidate genes underlying NDD and ASD. Determining the clinical significance of CNVs is sometimes challenging. To date, approximately 200 microdeletions and 80 microduplication syndromes have been described, and recurrent CNV is associated with a specific clinical picture [10,11,12,13]. The assessment becomes even more difficult in cases where there is no clear clinical picture and CNVs are found to contain genes of unclear clinical relevance. The challenge for the future lies in the possibility that multiple CNVs may be discovered in a patient that may have been inherited from “normal” parents. In this study, we used a gene-oriented, customized CMA to detect pathogenic/probably pathogenic CNVs in patients with NDD and ASD. In addition, this study focused on identifying and prioritizing new and previously uncharacterized candidate genes, integrating multiple levels of omics data to understand their potential contribution to NDDs and ASD.

## 2. Materials and Methods

### 2.1. Gene Selection and CMA Design Strategy

A gene-oriented microarray was developed specifically for the detection of single- and multi-exon CNVs. A customized microarray (“Custom CGH Zagreb”) was developed to examine 6026 genes with a high-density probe covering clinically relevant and candidate genes. This was achieved using Agilent’s SureDesign online platform with a total of 55,041 query probes and an average spacing of 8.2 kb (0.66–20 kb probe spacing) [14].

### 2.2. Experimental Procedure

Prior to CMA, Giemsa (GTG)-based band cytogenetics was performed on chromosomes from peripheral blood lymphocytes of the patient according to standard procedures. Genomic DNA was extracted from whole blood using the Puregene DNA Purification Kit (Gentra Systems, Minneapolis, MN, USA) according to the manufacturer’s protocol. The amount and purity of the DNA were determined using NanoDrop 2000, (Thermo Scientific, Kemolab, Croatia). Multiplex ligation-dependent probe amplification (MLPA) was performed according to the manufacturer’s instructions (MRC-Holland, Amsterdan, The Netherlands) using the ABI-PRISM 3130XL Genetic Analyzer (Applied Biosystems, Foster City, CA, USA). Data analysis was performed using the GeneMarker software package (SoftGenetics, PA, USA). The samples P070 subtelomeres, P036 subtelomeres, P245 microdeletion, ME028 Prader–Willi/Angelman, ME029 FMR1-AFF2, P106 X-chromosomal ID, P015 MECP2, and P064 microdeletion probemixes were used. CMA analysis was performed using SurePrint G3 4X180K CMA and custom CMA (Agilent, Santa Clara, CA, USA) according to the manufacturer’s instructions. Promega Human Genomic sex-matched non-disease DNA (Promega, Mannheim, Germany) was used as a reference. Data analysis was performed using CytoGenomics 4.0 software (Agilent, Santa Clara, CA, USA), and the GRCh38 assembly was used.

### 2.3. Sample Selection

To validate the microarray design, individuals who had previously undergone genetic screening in our laboratory using either MLPA or CMA and in whom structural variations had been confirmed were selected, in total, 9 cases. The patients were examined using a customized 8X60K and SurePrint G3 4X180K Agilent microarray. In addition, 30 patients with unexplained developmental delay/intellectual disability and/or ASD were included in this study and tested with Custom CGH Zagreb. Inclusion criteria for the customized CMA analysis were normal GTG banding and the absence of specific clinical dysmorphologies for microdeletion/microduplication syndromes.

### 2.4. Data Analysis

The identified CNVs were first evaluated for clinical relevance [8,15]. After excluding pathogenic and likely pathogenic CNVs in the remaining intervals, the following algorithm was applied:

- Separation of the altered regions into CNV losses and gains. The intervals from the two datasets were merged using bedtools MergeBED Galaxy Version 2.30.0. The merging was performed independently for loss and gain intervals [16].
- Overlapping intervals of loss and gain were subtracted using bedtools SubtractBed Galaxy Version 2.30.0. This resulted in intervals that were detected as both CNV loss and CNV gain in the tested samples [16].
- To detect unique CNV losses and gains, the bedtools Intersect intervals Galaxy Version 2.30.0 tool was used. This resulted in intervals that were detected in the tested samples only as copy number loss and copy number gain [16]. A complete list of CNV regions can be found in Supplementary Table S1.
- The genomic coordinates of the unique CNV loss and gain regions were used to retrieve genes from RefSeq Select+MANE and RefSeq Curated hg38, UCSC Table Browser. A custom CMA gene list was created from the RefSeq Select+MANE genes—MANE custom CMA gene list (Supplementary Table S2).
- The genomic coordinates of the unique CNV loss and gain regions were used in the web-based tool Ensembl BioMart to retrieve long non-coding RNAs. The search was restricted to “Gene type: lncRNA.” A complete list of all lncRNAs can be found in Supplementary Table S3.

### 2.5. Custom CMA Interactome

The entire human interactome was constructed using the Human Integrated Protein–Protein Interaction Reference (HIPPIE) database (version 2.3) [17]. Interactions with a confidence value of 0.63 and medium confidence were filtered out. As a result, a human interactome with 19,360 nodes/genes and 733,013 edges/interactions was designed. Next, protein–protein interactions for the MANE custom CMA gene list were extracted based on their first neighbors (Supplemental Table S4). The Cytoscape 3.9.1 software was used for network visualization and calculation of the centrality parameters of the networks [18].

### 2.6. Custom CMA NDD Interactome and Extending CMA Candidate Gene List

To filter out interactions specific to NDD genes from the entire custom CMA interactome, two independent sub-interactomes were created:

- A list of “NDD genes” was compiled to prioritize protein–protein interactions associated with NDDs. The “NDD gene panel” consists of genes selected from multiple web server sources, including SFARI Genes (Score 1, Score 2, and syndromic genes were included); the human brain proteome (detected in single genes: detected only in the brain); Synaptic Gene Ontologies genes; OMIM (search term “neurodevelopmental”); ENTREZ; gene2phenotype (search term “cognition,” confidence category: definite and strong); SysNDD database (category: definite). The integrative “NDD gene panel” can be found in Supplementary Table S5. Next, an integrative “NDD gene panel” list was used together with MANE’s custom CMA gene list to expand the selection of nodes (query nodes) using network propagation by the diffusion algorithm (v.1.6.1). Network propagation estimates network proximity (strength association between genes located in network proximity) and finds subnetworks in which the nodes are closely connected. Network diffusion aims to understand how changes in one part of the network can affect the entire network. The diffusion output value was set to 310. A resulting network with 824 nodes and 1221 edges is available in the Supplementary Table S6.
- Next, MANE’s custom CMA gene list was used as input for the interactome “Interaction network of proteins associated with intellectual disability and global developmental delay” in NDEx (v1.4) to extract significant associations between custom CMA genes and evolutionarily conserved and brain-expressed new putative candidates based on the MCL algorithm. Each node corresponds to a protein, and each line represents an interaction with high sustainability (STRING version 11.0; combined score > 0.9) [19]. A 1-step neighbor query yielded an output network with 207 nodes and 278 edges (Supplementary Table S7).

**Figure 1:**
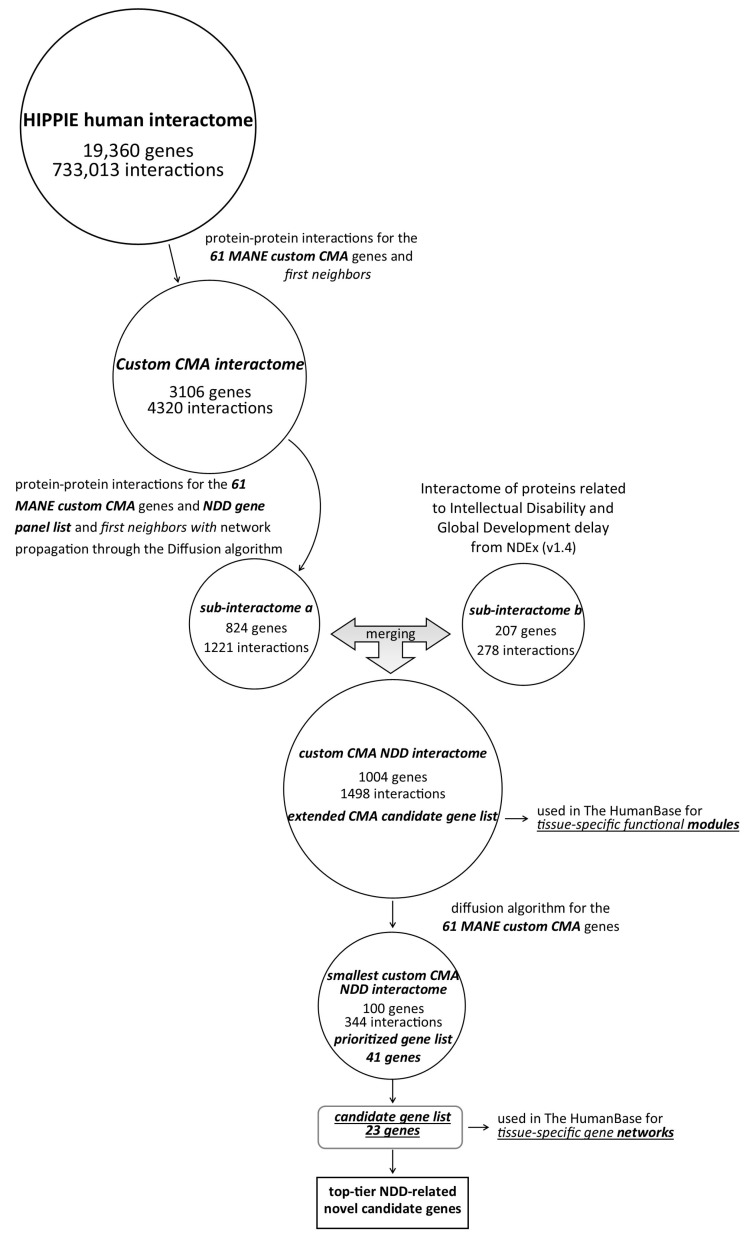
Schematic representation of the workflow.

The two resulting networks were merged, and a custom CMA-NDD interactome with 1004 nodes and 1498 edges was created. The expanded list of CMA candidate genes is shown in Supplementary Table S8. Cytoscape 3.9.1 software was used to visualize the network and calculate the centrality parameters of the networks. The workflow is summarized in Figure 1.

### 2.7. Tissue-Specific Functional Modules

The HumanBase database is a tool for discovering functional modules that facilitates network-based functional interpretation of genes and gene sets [20,21,22,23,24]. An extended CMA candidate gene list was used to extract significant biological processes using “Modules: functional module detection,” which allowed the discovery of tissue-specific functional modules containing genes of interest that are located in clusters sharing local network environments. In addition to a global network, networks were extracted for fetuses, embryos, the central nervous system, the brain, the cerebral cortex, neurons, glial cells, and astrocytes. Modules with custom CMA genes were selected from the resulting networks and the five most important GO terms per module were extracted.

### 2.8. Novel NDD Candidate Gene Prioritization

Next, a custom CMA gene list was used as a query to prioritize novel protein-coding NDD candidate genes from the custom CMA NDD interactome using the Diffusion algorithm (v.1.6.1). The diffusion output rank was set to 99. The resulting network was delineated as a full STRING network; confidence was set up to 0.4 with zero maximum additional interactors, thus presenting the smallest custom CMA NDD interactome (Figure 1) [25]. A complete list of genes presented in the smallest custom CMA NDD interactome can be found in Supplementary Table S9. The prioritized gen list, denoting a list of custom CMA NDD genes present in the smallest custom CMA NDD interactome, is available in Supplementary Table S10.

### 2.9. Novel NDD Candidate Gene Selection

To evaluate the contribution of prioritized genes to the pathophysiology of cognitive deficits in neurological development and to identify new NDD candidate genes, the following information was compiled:

- The probability predicted by pLI, LOEUF, sHet, pHaplo, and pTriplo was retrieved from Decipher.
- The Mouse Genome Informatics (MGI) database was searched for matching mouse models.
- Gene expression data were retrieved from the Human Brain Transcriptome database. In addition, protein, RNA, and single-cell expression data from the Human Protein Atlas were evaluated.
- The Database of Genomic Variants (Gold Standard) was searched for data on genomic variations in the general population.
- The PubMed databases were searched for comparable literature data.

After evaluating the information retrieved above, candidate genes were selected and tissue-specific gene networks were created in HumanBase using data from co-expression, protein interaction, TF binding, microRNA targets, and perturbation for embryo, fetus, global, central nervous system, brain, and cerebral cortex tissues. Future tissue-specific gene networks were created for neurons, glia, and astrocytes (Figure 1). The parameters used to create the networks were minimum interaction confidence > 0.10, maximum number of genes in the network set to 15, and the function “Prioritize by uniqueness relative to query genes” selected. After evaluating all data obtained from the above sources, a list of the most important new NDD-related candidate genes was created.

## 3. Results

### 3.1. Custom CMA Validation and Pathogenic and Likely Pathogenic CNVs Detected by Custom CMA

The results that passed quality control for 180 K and 60 K CMA were evaluated to validate the CMA design. As a result, pathogenic and likely pathogenic CNVs were detected in five cases with both 180 K and customer-specific 60 K CMA (Supplementary Table S11). Of the samples tested only with custom CMA, 29 samples showed excellent quality control metrics, and 1 case failed to pass the quality control metrics. Pathogenic and likely pathogenic aberrations were detected in nine patients, corresponding to a diagnostic yield of 31% (9/29) (Supplementary Table S12).

### 3.2. Interactomes—From the Whole Human Interactome to the Smallest Custom CMA NDDi

The average centrality measures were calculated for all interactomes (Supplementary Table S13). The average value is calculated for intermediate centrality and degree to minimize outliers, while the median value is calculated for shortest path length and proximity.

Extraction of the custom CMA gene list and its first neighbors from the HIPPIE human interactome resulted in a custom CMA interactome with 3106 nodes/genes and 4320 edges/interactions. Three genes had only one interaction, while one gene had four interactions and was displayed independently of the custom CMAi.

**Figure 2:**
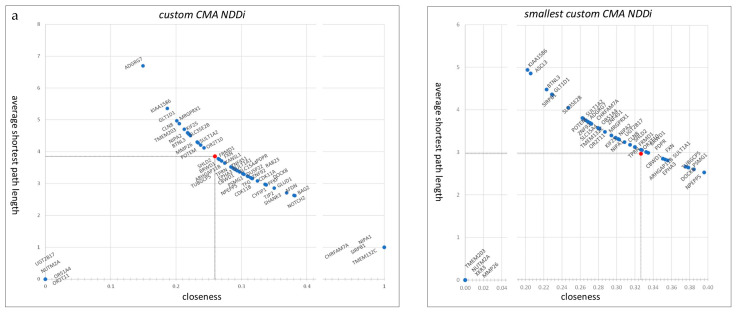

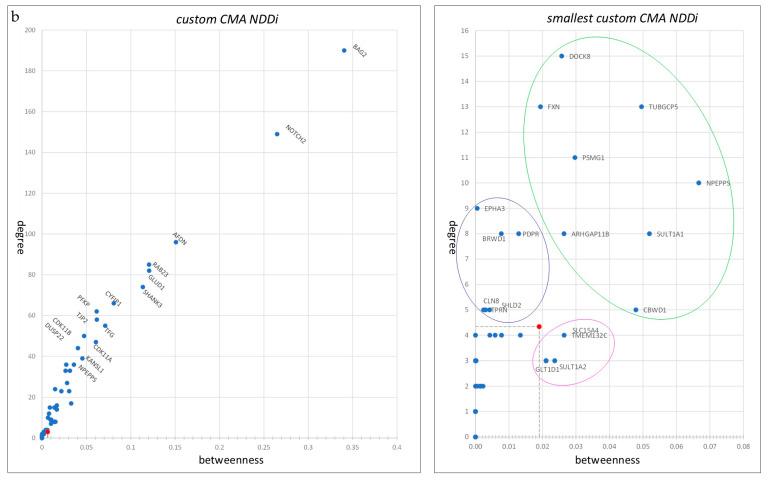
Centrality parameters of the custom and smallest CMA NDDi networks. (**a**) Distribution of closeness centrality and shortest path length. The median values for both the custom and smallest CMA NDDi networks are indicated by red dots. (**b**) Measurement of centrality using the ratio of betweenness to degree in the custom and smallest CMA NDDi networks. Median centrality values are shown as red dots. Within the smallest custom CMA NDDi, three distinct patterns based on centrality parameters are evident and are highlighted by circles. First are genes with higher centrality values, including *NPEPPS*, *PSMG1*, *DOCK8*, *TUBGCP5*, *SULT1A1*, *FXN*, *ARHGAP11B*, and *CBWD1* (encircled in green). The second is higher betweenness and low-degree genes, including *TMEM132C*, *SLC15A4*, *SULT1A2*, and *GLT1D1* (encircled in magenta). The third pattern genes with a higher degree but lower betweenness, including *EPHA3*, *PDPR*, *BRWD1*, *TPRN*, *SHLD2*, and *CLN8* (encircled in blue).

Furthermore, a downstream analysis narrowed down the gene selection. As a result, the custom CMA NDDi was created, containing 1004 nodes/genes and 1498 edges/interactions, including 55 genes from the custom CMA list and 782 genes from the “NDD gene panel.” Of the 55 genes, 13 were found in the “NDD gene panel” (Supplementary Table S14). In the custom CMA NDDi, five genes are represented as single nodes, while four genes have only one interaction, and one gene has two interactions. All of these genes were introduced independently of the custom CMA NDDi. Taking into account the shortest path length and proximity measurements, 22 genes showed high proximity and lower values for the shortest path length compared to the median of the custom CMA NDDi. In contrast, 14 genes showed lower proximity and higher shortest path length than the median of the custom CMA NDDi (Figure 2a). In addition, more than half of the custom CMA genes showed higher centrality values (betweenness/degree) compared to the average of the complete custom CNV NDDi (Figure 2b). In this sense, 36 custom CMA genes could be considered hubs, in particular *BAG2*, *NOTCH2*, and *AFDN* with degrees of 190, 149, and 96, respectively.

**Figure 3:**
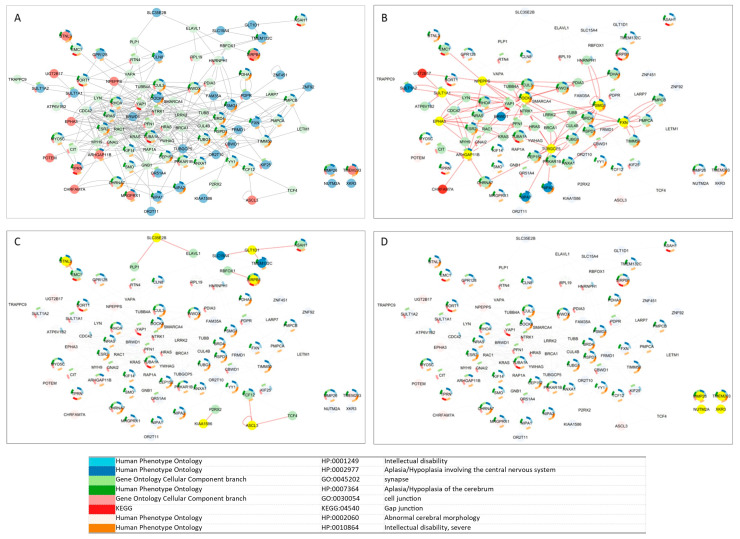
Smallest custom CMA NDDi network. (**A**) Candidate genes derived from CNV loss are shown in red, while those from the CNV gain group are shown in blue. (**B**) Candidate genes exhibiting high closeness centrality and reduced shortest path length relative to the median of the smallest custom CMA NDDi are highlighted in yellow. (**C**) Candidate genes characterized by low closeness centrality and elevated average shortest path length are indicated in yellow. (**D**) Candidate genes presented as singletons, with no network connections, are marked in yellow. Gene enrichment analysis was performed with Cytoscape 3.9.1 software.

In the smallest custom CMA NDDi, 41 genes from the custom CMA list and 68 from the NDD list were present (Figure 3A). Of the 41 genes, 9 were included in the NDD list.

Considering the shortest path length and proximity measurements, nine genes showed high proximity and lower values for the shortest path length compared to the median of the smallest custom CMA NDDi, including *NPEPPS*, *PSMG1*, *DOCK8*, *TUBGCP5*, *SULT1A1*, *FXN*, *EPHA3*, *ARHGAP11B*, and *CBWD1* (Figure 2a,b). In contrast, *KIAA1586*, *ASCL3*, *BTNL3*, *SIRPB1*, and *GLT1D1* show low proximity and high average shortest path length (Figure 2a,b). Only four nodes were represented as singletons (Figure 3D).

The centrality parameters (betweenness/degree) can identify three distinct patterns. The first pattern includes genes with higher centrality values, including *NPEPPS*, *PSMG1*, *DOCK8*, *TUBGCP5*, *SULT1A1*, *FXN*, *ARHGAP11B*, and *CBWD1*. The second pattern includes genes with higher intermediate centrality and low degree, including *TMEM132C*, *SLC15A4*, *SULT1A2*, and *GLT1D1*. The third pattern includes genes with a higher degree but lower intermediate centrality, including *EPHA3*, *PDPR*, *BRWD1*, *TPRN*, *SHLD2*, and *CLN8* (Figure 2b).

### 3.3. Extended Custom CMA List Within Tissue-Specific Network-Based Functional Modules

To gain insights into the biological context of expanded custom specific CMA genes, tissue-specific network-based functional modules were created using the expanded CMA gene list from The HumanBase (Supplementary Table S15). Taking into account the spatiotemporal function of the genes, the tissue networks and modules were divided into the prenatal group (embryo and fetus), the postnatal tissue group (global, central nervous system, brain, and cerebral cortex), and the postnatal cell group (neurons, glial cells, and astrocytes). Modules containing custom CMA genes were extracted from the resulting networks. Of the 56 custom CMA genes, 55 genes were present in modules. The largest number of genes was found in embryonic tissue (46 genes), while the smallest number was found in astrocyte tissue (33 genes). Several modules had specific genes. In Neuron-M2, *XKR3* is followed by *OR2T10*, *OR2T11*, and *OR51A*, which occur only in Embryo-M5. Next, *ARHGAP11B*, which occurs only in global-M4 and glia-M3, and *CDK11B*, which occurs only in cerebral cortex-M4 and glia-M2. In contrast, 24 genes occur in eight or nine modules (Supplementary Table S15).

Considering specific time windows during prenatal brain development, embryonic and fetal tissue-specific networks share 40 genes. *GLT1D1*, *KIAA1586*, and *TPRN* were exclusive to the fetal network, whereas *NIPA1*, *SHANK3*, *NUTM2A*, *OR2T10*, *OR2T11*, and *OR51A4* were identified in embryonic tissue-specific networks (Figure S1).

In general, when comparing tissue-specific network modules from the global network across the central nervous system, the brain, and the cerebral cortex, most genes are involved in more than three modules. Specific individual genes that appeared in only one module were *CDK11B*, *CLN8*, *DUSP22*, and *CBWD1* in the cerebral cortex network and *POTEM* and *ARHGAP11B* in the global network (Figure S2). To better understand the influence of each gene at the cellular level, neuron-, glia-, and astrocyte-specific networks were evaluated. Most genes were involved in all three modules, namely 25 genes. Specific genes in the neuro module were *CDK11A*, *XKR3*, *ADGRG7*, *ZNF451*, and *SULT1A2*; in the glia module, *MRGPRX1*, *CDK11B*, *ARHGAP11B*, and *TFG*; and in the astrocyte module, *GLUD1* (Figure S3).

Forty-seven modules were filtered from nine tissue networks, and the top five GO terms for each module were extracted. Out of a total of 235 GO terms, 123 were unique. More than half, specifically 71 GO terms, are linked to only one module, whereas the neurogenesis GO:0022008 is associated with eight modules (Supplementary Table S16). Twenty-five GO terms are linked solely to the astrocyte module, while the neuron module contains ten unique GO terms. The global and fetus models have nine unique GO terms, while the embryo module has eight. The glia and cerebral cortex modules each have three unique GO terms, and the CNS and brain modules have only two unique GO terms (Supplementary Table S17).

### 3.4. NDD Candidate CNVs

Of the 62 custom CMA genes, 41 were present in the smallest custom NDD interactome with 100 nodes/genes—prioritized genes. Nine of the forty-one genes are included in the NDD list: *NIPA2*, *NIPA1*, *KIAA1586*, *OR2T10*, *TUBGCP5*, *ARHGAP11B*, *CLN8*, *DOCK8*, and *EPHA3*. Among the prioritized genes, *NIPA2*, *TMEM132C*, and *TMEM203* had the highest pLI scores. *SLC35E2B*, *FRMD1*, and *BRWD1* had pTriplo scores ≥ 0.68, while *TMEM203* and *NPEPPS* had pHaplo scores ≥ 0.55 (Supplementary Table S10).

The MGI database does not contain any associated genes for *KIF25*, *MMP26*, *FRMD1*, *OR2T11*, *OR51A4*, *ZNF92*, *KIAA1586*, and *OR2T10*. A total of 344 mammalian phenotypes were reported for the prioritized genes (Supplementary Table S18). No abnormal phenotype was found for *ASCL3*. The ten most frequently reported mammalian phenotypes (MP) are abnormal spleen morphology MP:0000689, abnormal skin morphology MP:0002060, enlarged spleen MP:0000691, hyperactivity MP:0001399, decreased motor activity MP:0001402, male infertility MP:0001925, abnormal heart morphology MP:0000266, enlarged heart MP:0000274, abnormal testicular morphology MP:0001146, and reduced body weight MP:0001262 (Supplementary Table S18).

No gene expression data are available for 14 genes in the Human Brain Transcriptome Database. Among the prioritized genes, *ZNF451*, *SLC15A4*, *NPEPPS*, *PSMG1*, and *BRWD1* show the highest stable expression throughout the lifespan. *EPHA3*, *KIAA1586*, *NIPA2*, *ZNF92*, and *FXN* are highly expressed in the prenatal human brain, with expression decreasing after birth. This decrease is particularly pronounced in *EPHA3*. In contrast, *SULT1A1*, *NIPA1*, *GLT1D1*, and *CLN8* show increased expression from the embryonic stage to the neonatal phase, when expression becomes more stable. The Human Protein Atlas shows that protein expression levels among the priority genes do not exhibit a specific pattern. At the RNA tissue level, *KIF25* and *TPRN* show increased expression in the brain, while *TMEM132C*, *GLT1D1*, *NIPA1*, and *BRWD1* show stronger expression in CNS tissue. At the single-cell level, *EPHA3*, *CLN8*, *DOCK8*, *TMEM132C*, *SLC15A4*, *GLT1D1*, and *CHRFAM7A* are enhanced or enriched in various CNS cells.

The genomic variation database contains no entries for *ZNF92*, *CLN8*, *TPRN*, *PSMG1*, and *BRWD1*. For most candidate genes, only minor structural variations are present (Supplementary Table S19, Figure S4). Furthermore, most of the structural variations identified in candidate genes have an internal rank above 7. Only gssvL111523 (chr6:168075918-168075918) has an internal rank of 1, gssvL128976 (chr9:322405-322405) and gssvL130466 (chr9:69050657-69050657) have an internal rank of 2, and gssvL82637 (chr3:89466937-89466937) has an internal rank of 3.

Based on the retrieved data, 16 CNV candidates were selected, including 23 MANE-selected transcripts (Supplementary Table S20). Gene expression data are summarized for 23 protein-coding genes from the CNV candidates (Supplementary Table S21).

### 3.5. Tissue-Specific Gene Networks for Protein-Coding Genes from Candidate CNVs

**Figure 4:**
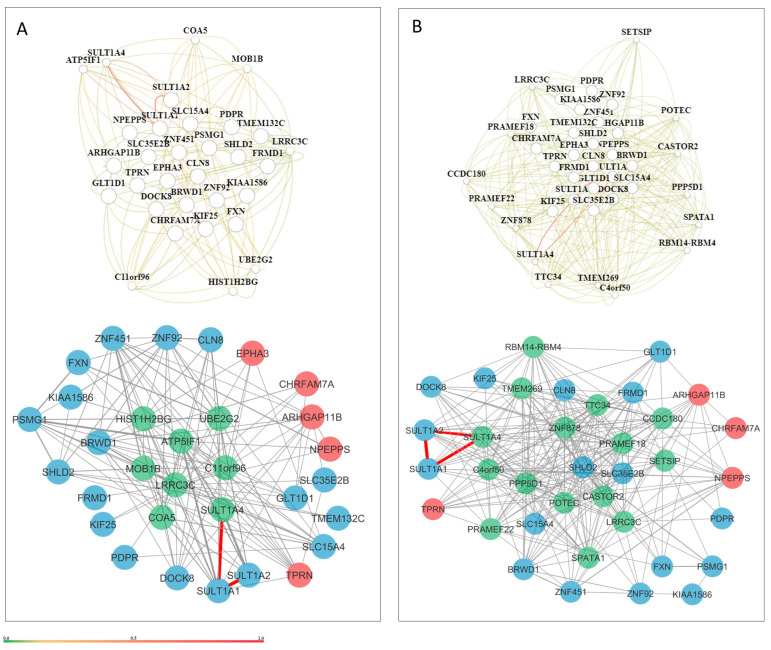
Tissue-specific gene networks for NDD candidate genes from HumanBase. (**A**) Fetus-specific network and (**B**) embryo-specific network. Gene interactions with confidence scores greater than 0.5 are shown in dark red, including interactions involving *SULT1A2*, *SULT1A1*, and *SULT1A4*. A confidence scale bar is displayed below the network. Candidate genes derived from CNV loss are shown in red, while those from the CNV gain group are shown in blue.

**Figure 5:**
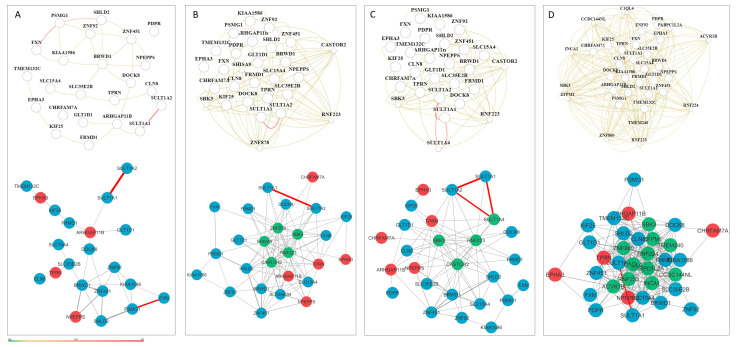
Tissue-specific gene networks for NDD candidate genes from HumanBase for (**A**) global network, (**B**) central nervous system, (**C**) brain, and (**D**) cerebral cortex. Gene interactions with confidence scores greater than 0.5 are shown in dark red, including interactions involving *SULT1A2*, *SULT1A1*, and *SULT1A4*. A confidence scale bar is displayed below the network. Candidate genes derived from CNV loss are shown in red, while those from the CNV gain group are shown in blue.

**Figure 6:**
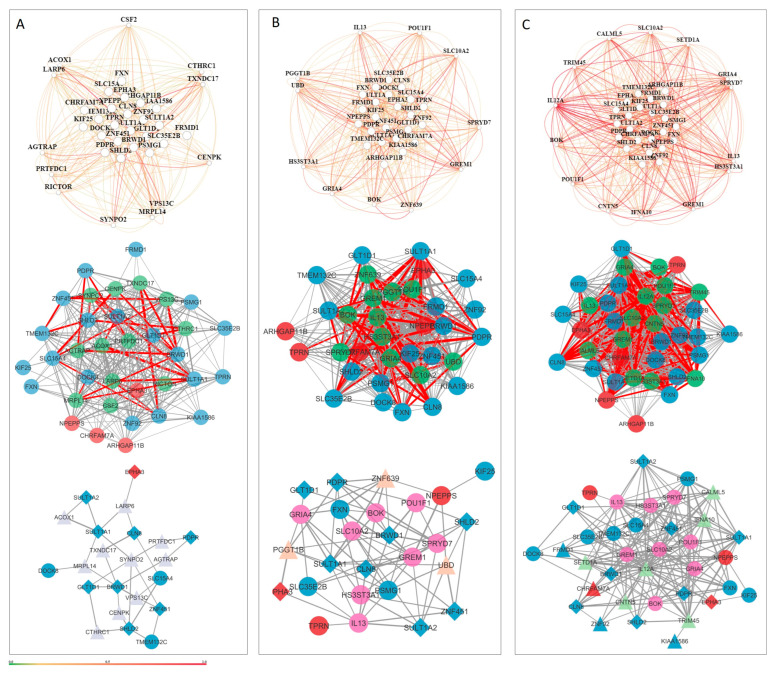
Tissue-specific gene networks for NDD candidate genes from HumanBase. Gene networks are shown for three brain cell types: (**A**) neurons, comprising 22 genes and 31 interactions; (**B**) glial cells, comprising 26 genes and 77 interactions; and (**C**) astrocytes, comprising 36 genes and 181 interactions. Red circles represent candidate genes originating from CNV loss, while those from CNV gain are shown as blue circles. Additional cell-type-specific genes incorporated into the networks are depicted as triangles. Candidate genes present in all three networks are indicated by a diamond shape. Genes shared between the astrocyte and glial networks are highlighted in magenta. Edge thickness corresponds to interaction strength, with thicker edges indicating higher confidence in gene–gene interactions.

Tissue-specific gene networks for NDD candidate genes were extracted from HumanBase, and only interactions above 0.5 were considered for further evaluation, reflecting stronger evidence of the biological basis of the interaction. Candidate genes show interactions during the prenatal phase, but with low interaction certainty (Figure 4). Gene interaction certainty above 0.5 is observed for *SULT1A2*, *SULT1A1*, and *SULT1A4* genes. The same pattern of low certainty is also evident at the tissue level (Figure 5). Candidate genes show complex and strong interactions with high certainty only in the cell-level networks. For the neuronal cell network, 22 genes/31 interactions (Figure 6A), for the glial cell network, 26 genes/77 interactions (Figure 6B), and for the astrocyte cell network, 36 genes/181 interactions (Figure 6C) show interaction confidence above 0.5. Candidate genes with an interaction confidence above 0.5 that are present in all three cell type networks are *PDPR*, *CLN8*, *BRWD1*, *SHLD2*, *GLT1D1*, *SULT1A1*, *ZNF451*, *SULT1A2*, and *EPHA3*. The neuronal network has unique interacting genes (Figure 6A), while the glia and astrocyte networks share eight interacting genes, including *POU1F1*, *IL13*, *BOK*, *GRIA4*, *GREM1*, *SPRYD7*, *HS3ST3A1*, and *SLC10A2* (Figure 6). Specific candidate genes for the astrocyte network are *ZNF92*, *KIAA1586*, *FRMD1*, and *CHRFAM7A* (Figure 6B).

### 3.6. Top-Tier NDD-Related Novel Candidate CNVs in Patient Context

Of the candidate CNVs, five NDD candidate regions have already been identified, including 3p11.1 (*EPHA3*), 6p12.1 (*KIAA1586*), 8p23.3 (*CLN8*), 9p24.3 (*DOCK8*), and 15q13.2 (*CHRFAM7A*, *ARHGAP11B*). Based on the combined analysis steps and interpretation of the collected data, the most likely candidates for copy number loss are the genes *TPRN* and *NPEPPS*, while the most likely candidates for copy number gain are the genes *ZNF451*, *KIF25* and *FRMD1* from 6q27; *ZNF92*, *FXN*, *TMEM132C*, *SLC15A4*, and *GLT1D1* from 12q24.32q24.33; and *PSMG1* and *BRWD1* from 21q22.2

## 4. Discussion

### 4.1. Custom CMA Validation and Diagnostic Yield

CMA is a diagnostic test with a documented yield in the postnatal evaluation of patients with unexplained developmental delay, intellectual disability, congenital anomalies, and ASD, generally ranging from 5% to 20%. Factors such as the microarray platform, patient population, and interpretation criteria may influence the yield. This study utilized commercial 180 K and custom 60 K Agilent platforms. The custom 60 K array detected pathogenic and likely pathogenic aberrations in all patients identified with the commercial 180 K array. Furthermore, pathogenic and probable pathogenic aberrations were found in nine patients using the customized 60 K array, resulting in a diagnostic yield of 31%. This above-average diagnostic yield could stem from the gene-oriented design of the customized array.

### 4.2. Interactome Analysis

First, MANE’s custom CNV genes were evaluated within an interactome gene network, progressing from the entire human interactome to the smallest custom CMA-NDD interactome. The calculated average centrality measures included proximity, average shortest path length, degree, and betweenness. Regarding prioritized custom CMA genes within the smallest custom CMA-NDDi, several genes exhibited higher average proximity and lower shortest path lengths. These genes include NPEPPS, PSMG1, DOCK8, and *TUBGCP5*, followed by *FXN*, *ARHGAP11B*, *SULT1A1*, *CBWD1*, and *EPHA3* (Figure 2a,b). In gene networks, proximity centrality and average shortest path length indicate the accessibility and integration of genes within the network. Proximity centrality assesses the average distance of a gene to all other genes within the network, with genes closer to others having higher proximity centrality. Genes with high proximity centrality are considered more central and integrated within the network, enabling efficient information and signal exchange with other genes. The average shortest path length measures the average distance between all gene pairs in the network. A smaller average shortest path length signals a more interconnected and integrated network, where genes can easily exchange information and signals. Genes that demonstrate high proximity centrality and low average shortest path length are likely to be critical in regulating cellular processes, as they are well-connected and can swiftly share information with other genes. This enables these genes to react promptly to changes in the cellular environment and coordinate complex processes. Furthermore, genes displaying high proximity centrality and low average shortest path length may compensate for the functional loss of other genes. In this context, *NPEPPS*, *PSMG1*, *DOCK8*, and *TUBGCP5* could play significant roles in the smallest custom CMA NDDi.

In contrast, genes with low proximity centrality and high average shortest path length may be less connected and less involved in regulating cellular processes. They may be more vulnerable to genetic disorders. Among the prioritized custom CMA genes in the smallest custom CMA NDDi, *KIAA1586*, *ASCL3*, *BTNL3*, *SIRPB1*, and *GLT1D1* exhibit high average shortest path length and low proximity, indicating vulnerability to genetic disorders (Figure 2a).

On the other hand, specific patterns of degree and intermediate centrality in the smallest custom CMA NDDi are illustrated for the custom CMA genes (Figure 2b). In a biological network, degree centrality refers to the number of connections of a given node within the network. Nodes with higher degree values are considered hubs and may have multiple functions in cellular networks. Intermediate centrality describes a node’s influence over the flow of information in the network, corresponding to the number of non-redundant shortest paths that pass through it. This can indicate the protein’s potential to serve as a bridge for communication between distant nodes. There are three distinct patterns in the smallest CMA NDDi. First are genes with a high degree and higher intermediate centrality. Genes exhibiting both high degree and high intermediate centrality in a biological network are “hubs” that play a crucial role in the structure and function of the network, such as *DOCK8*, *FXN*, *TUBGCP5*, *PSMG1*, *NPEPPS*, *ARHGAP11B*, *SULT1A1*, and *CBWD1*.

Interestingly, EPHA3, BRWD1, PDPR, CLN8, TPRN, and SHLD2 exhibit an above-average degree but low betweenness centrality. This suggests that while they have numerous connections to other genes, they do not play a crucial role in the transmission of information between genes. In a biological network, these genes are referred to as “provincial hubs,” possibly indicating their greater involvement in local interactions and regulatory processes rather than serving as mediators between distant genes in the network. In biological systems, such genes can significantly influence the regulation of cell processes confined to specific tissues or cell compartments. They might also contribute to the regulation of more specialized processes, which is crucial in the context of disease associations. Mutations or dysregulation in provincial hub genes may correlate with diseases, especially those involving localized cell dysfunction or specific tissue types. Due to their specialized functions, these genes may be vital in disease contexts where these functions are critical.

Finally, genes with a low degree and higher intermediate degree, such as *SLC15A4*, *TMEM132C*, *GLT1D1*, and *SULT1A2*, are found in the smallest custom CMA NDDi, within a biological network known as “connector” or “bottleneck” genes. They have relatively few direct connections to other genes in the network but play a crucial role in facilitating communication and information flow among different parts of it. Interestingly, *SLC15A4*, *TMEM132C*, and *GLT1D1* are located in the same CNV region (12q24.32q24.33) and were identified as CNV gains in one patient. Furthermore, they are notable for study due to their diverse biological background, as disruption of these genes in the context of NDD may lead to impaired signal transmission and coordination. Genes with a low degree but high intermediate centrality also mediate information flow between network components. They are strategically positioned to transfer information between groups of genes that may not be directly linked, integrate signals from multiple signaling pathways, and relay them to the appropriate downstream targets. Connector genes can function as regulatory hubs that control and modulate the activities of other genes, even though they do not engage in many direct interactions. They may be involved in important regulatory processes such as transcription regulation, post-translational modifications, or signal transduction.

Table 1 provides a summary of gene roles based on centrality measures (proximity, shortest path length, degree, and betweenness) in the smallest custom CMA-NDDi interactome.

In summary, the analysis of the average centrality measures of the smallest custom CMA-NDDi network revealed that *DOCK8*, *FXN*, *TUBGCP5*, *PSMG1*, *NPEPPS*, *ARHGAP11B*, *SULT1A1*, and *CBWD1* from the prioritized gene list can be considered “hubs.” Mutations or dysregulation in “hub” genes can have profound effects on the entire network, leading to disease. Next, based on average centrality measures, *EPHA3*, *BRWD1*, *PDPR*, *CLN8*, *TPRN*, and *SHLD2* from the prioritized gene list could be viewed as “provincial hubs” that are important for specific, localized functions within the network. They may have numerous direct interactions with other genes, but do not play a central role in connecting different parts of the network. Finally, *SLC15A4*, *TMEM132C*, *GLT1D1*, and *SULT1A2* are located in the smallest custom CMA NDDi within a biological network, referred to as “connector” or “bottleneck” genes, and they play a key role in coordinating and relaying information within biological networks. Although they may not have many direct connections, their strategic positioning makes them essential for the proper functioning and regulation of complex cellular processes.

### 4.3. Extended Custom CMA List Within Tissue-Specific Network-Based Functional Modules

To elucidate the biological relevance of expanded customer-specific CMA genes, we utilized tissue-specific, network-based functional modules from HumanBase. By stratifying the networks according to developmental time points and cellular contexts, our approach facilitated a systematic analysis of gene functions across prenatal and postnatal stages and within tissue- and cell-specific contexts.

Our results indicate a high integration of custom CMA genes into functional modules, with 55 out of 56 genes present in at least one module. The embryonic tissue-specific network encompassed the largest subgroup of CMA genes (n46). In contrast, fewer genes were identified in astrocyte-specific modules (33), possibly indicating more specialized or restricted functions.

Several genes demonstrated strict module-specific localization, indicating highly context-specific functions. For instance, XKR3 was uniquely enriched in the neuron-M2 module, while *OR2T10*, *OR2T11*, and *OR51A4* were exclusively located in the embryonic M5 module.

Considering specific prenatal time periods, the overlap between fetal and embryonic tissue-specific networks was significant, with 40 genes in common. However, distinct differences were also noted. *NIPA1*, *SHANK3*, and olfactory receptor genes were limited to the embryonic stage, while *GLT1D1*, *KIAA1586*, and *TPRN* were exclusive to the fetal stage, suggesting a nuanced temporal regulation of gene function during early brain development.

When examining tissue specificity in the postnatal phase, most genes tended to integrate into multiple modules, suggesting largely conserved functional roles. However, unique associations with a single module, such as *CDK11B*, *CLN8*, *DUSP22*, and *CBWD1* in the cerebral cortex network, and *POTEM* and *ARHGAP11B* in the global network, indicated specialized functional relevance.

At the cellular level, 25 genes were common among neuron, glia, and astrocyte modules, illustrating broad functional integration. Conversely, distinct cellular associations were also identified: *CDK11A*, *XKR3*, and *SULT1A2* were neuron-specific, *MRGPRX1*, *TFG*, and *ARHGAP11B* were specific to glial cells, and *GLUD1* was exclusively present in the astrocyte module, supporting the concept of cell-type-specific specialization. *GLUD1* single-cell expression data confirm the astrocyte-specific function, as astrocytes are enriched in cell types according to RNA brain cluster specificity [26].

Next, functional enrichment analyses of 47 filtered modules across nine networks yielded 235 GO terms, with 123 unique terms identified. Neurogenesis (GO:0022008) was recognized as a central biological process across eight modules, underscoring its fundamental role in CMA gene function. Astrocyte modules contributed the highest number of unique GO terms (n = 25), indicating a previously underestimated diversity of astrocyte gene functions. This finding is surprising, considering that the astrocyte module has a lower number of genes and fewer specific genes according to the analysis (Table S11 and Figure S3). This was followed by the neuron module (10 unique terms) and developmental stages (embryo, fetus, and global) with 8–9 unique terms each. In contrast, the glia, cerebral cortex, CNS, and brain modules displayed only limited uniqueness in their GO terms.

Table 2 provides a summary based on the analysis of the extended custom CMA gene list within tissue-specific, network-based functional modules.

These results highlight the complex, temporally dynamic, and context-dependent roles of custom CMA genes throughout brain development and function. The presence of distinct genes and GO terms in early development modules and cell-specific modules indicates that the expanded CMA gene set significantly contributes to neurological development processes and may affect susceptibility to developmental disorders.

### 4.4. NDD Candidate CNVs

Interestingly, 12 of the 16 CNV candidate regions represent gains, while only 4 are deletions, encompassing a total of 23 protein-coding candidate genes (Table S5). These genes demonstrate various expression patterns and phenotypic effects, highlighting the importance of multi-omics integration for prioritizing biologically relevant candidates for future functional validation. In particular, the convergence of dosage sensitivity, prenatal expression, and CNS-specific enrichment supports the hypothesis that these genes are likely to contribute to neurological developmental disorders associated with CNV regions.

### 4.5. Tissue-Specific Gene Networks for NDD Candidate Genes

To elucidate the functional relevance of the NDD candidate genes, tissue- and cell-type-specific interaction networks were analyzed using data from HumanBase. For the subsequent analysis, a confidence cutoff was set at >0.5. Lower-confidence interactions were excluded to ensure that the gene–gene interactions included reflected a higher degree of biological support. This threshold highlighted notable connection patterns, particularly within cell-type-specific networks.

Interestingly, candidate genes demonstrated relatively few reliable interactions within prenatal tissue networks, suggesting that co-functional relationships during early brain development may be transient or context-specific. Exceptions included *SULT1A1*, *SULT1A2*, and *SULT1A4*, which formed stable, highly reliable interactions (>0.5) (Figure 4). Some evidence suggests that SULT1A1 is highly expressed in early human brain development (between 4 and 17 weeks after conception), whereas the expression of *SULT1A2* is more restricted or lower in comparison [27]. *SLC26A1* and *SLC13A1* are two sulfate transporters that have been implicated in sulfate homeostasis and neurological disorders [28,29]. Variants in these genes have been associated with skeletal phenotypes, including short stature, scoliosis, and skeletal dysplasia. Additionally, SLC26A2-mediated sulfate metabolism has been shown to be important in tooth development [29].

At the tissue level, interaction remained moderate across various postnatal brain structures (e.g., cerebral cortex, global brain, CNS), indicating a potentially diffuse or modular pattern of gene activity that does not heavily depend on closely interconnected networks (Figure 5). The most robust and intricate gene interaction patterns were observed at the cellular level. In the neuronal network, 22 candidate genes established 31 highly reliable interactions. The glial network demonstrated an even higher interaction density (26 genes, 77 interactions), highlighting the increasingly recognized role of glial cells in neurodevelopment and synapse maintenance. Eight genes were shared within the astrocyte network, including *GRIA4* and *BOK.* This overlap indicates common glial mechanisms that may contribute to NDDs by modulating inflammation, synaptic pruning, or homeostasis. The astrocyte network was the most highly connected, with 36 genes forming 181 highly reliable interactions. The complexity of this network suggests that astrocytes may serve as important mediators of gene interaction networks in NDDs. Astrocyte-specific candidate genes included *ZNF92*, *FRMD1*, *KIAA1586*, and *CHRFAM7A*—genes associated with transcriptional control, structural integrity, and cholinergic signaling.

Nine candidate genes—*PDPR*, *CLN8*, *BRWD1*, *SHLD2*, *GLT1D1*, *SULT1A1*, *ZNF451*, *SULT1A2*, and *EPHA3*—were identified in all three cell-type-specific networks, indicating broad functional relevance across different neuronal cell types. These genes may serve as key regulators or vulnerabilities in the pathophysiology of NDDs due to their pleiotropic effects on neural circuit development, cellular homeostasis, and synaptic communication.

In Table 3, a summary of the tissue-specific gene networks of NDD candidate genes based on cell- and tissue-level interaction patterns is presented.

### 4.6. Top-Ranking NDD Candidate CNVs in Patient Context

New CNVs were discovered in seven cases based on the combined analysis steps and interpretation of the collected data (Table 4). Only one patient (Case 37) was found to have a pathogenic/probably pathogenic CNV (Table S2).

Case 10—A female patient exhibited severe multiple morphological anomalies, including microcephaly. At the time of diagnosis, she was completely immobile and unable to communicate. A duplication of the genes *PSMG1* and *BRWD1* from 21q22.2 was detected (Table 4). The *PSMG1* gene (proteasome assembly chaperone 1; HGNC:3043) encodes a chaperone protein that facilitates the assembly of the 20S proteasome as part of a heterodimer with *PSMG2*. This *PSMG1-PSMG2* heterodimer interacts with the *PSMA5* and *PSMA7* proteasome subunits, supporting the assembly of the proteasome alpha subunits [30,31,32]. The proteasome critically regulates gene expression and silencing through both proteolytic and non-proteolytic functions [33]. While *PSMG1* has not been directly linked to common NDDs, dysfunction of proteasome-associated genes such as *PSMB1* and *PSMC3* has been associated with autism, developmental delays, and microcephaly [34,35]. The gene *BRWD1* (bromodomain and WD repeat domain 1, HGNC:12760) is a transcription regulator involved in chromatin remodeling [36]. Furthermore, *BRWD1* plays a role in regulating cell morphology and cytoskeletal organization and is required for controlling cell shape (BRWD1_HUMAN, Q9NSI6). Both genes exhibit increased expression in all brain regions throughout life, with *PSMG1* showing a slight decrease between the early and middle fetal stages. According to interactome analysis, *PSMG1* functions as a central hub, while *BRWD1* may serve as a more specialized “provincial” hub (Figure 2b). This is supported by the observation that *BRWD1* is present in all three cell type networks, including neuronal, glial, and astrocyte-specific networks; this could indicate a neuronal cell-type-specific hub. RNA expression data for *BRWD1* suggest that it is clustered in neuronal tissue—primarily synaptic function (Table S6). CNV gain at 21q22.2, which includes the genes *PSMG1* and *BRWD1*, may be clinically significant, as no CNV for these specific genes has been reported in DGV, and pTriplo for the gene *BRWD1* is 0.89 (Table S15).

Case 14—A male patient was primarily affected by neurological developmental disorders. A deletion was detected at 9q34.3, impacting the gene *TPRN* (taperin, HGNC:26894). *TPRN* encodes a sensory epithelial protein. Mutations in *TPRN* are associated with a form of autosomal recessive non-syndromic deafness [37]. The functional annotation predicts an association of *TPRN* with myelination of the central nervous system (GO:0022010) and axon sheathing in the central nervous system (GO:0032291), which aligns with its RNA cluster specificity in the brain, indicating cell-type-specific expression in oligodendrocytes (Table S6) [38,39]. Furthermore, according to interactome analysis, *TPRN* is confined to the fetal network and may function as a “provincial” hub. No CNV is reported for this gene in the DGV gold standard.

Case 18—A male patient presented with distinctive facial features and neurological developmental disorders, including epilepsy. A duplication was detected at 7q11.21, affecting part of the gene *ZNF92* (zinc finger protein 92, HGNC:13168). *ZNF92* functions as a DNA-binding transcription factor with RNA polymerase II-specific and cis-regulatory sequence-specific DNA binding activity (Alliance of Genome Resources, June 2025). It is also thought to be involved in the regulation of DNA-templated transcription and localization to the cell nucleus (Alliance of Genome Resources, March 2025). This gene is highly expressed during prenatal brain development and shows a sharp decline after birth (Table S6). No CNVs for the gene *ZNF92* are reported in the DGV gold standard database. Interactome analysis specifically assigns the gene *ZNF92* to the astrocyte network.

Case 23—A female patient was diagnosed with neurodevelopmental disorders, cerebral atrophy, and pronounced dental anomalies. Two gene duplications were identified: *ZNF451* on 6p12.1 and *FXN* on 9q21.11. The gene *ZNF451* (zinc finger protein 451, HGNC:21091) is a multifunctional protein with SUMO ligase and transcriptional co-repressor activities. It plays a key role in DNA repair by dissolving TOP2 DNA complexes and activating *TDP2* [40,41]. *ZNF451* localizes in PML bodies and regulates transcription through SUMOylation and interactions with factors such as Smad3/4 and the androgen receptor [42]. It also influences cancer progression and therapy resistance, including in pancreatic, breast, and lung cancer [43,44,45]. *ZNF451* shows increased dynamic expression in all brain regions and developmental stages, with slight declines between early and late mid-fetal development (Table S6). In contrast, single-cell RNA type specificity indicates that the expression of *ZNF451* is particularly enhanced in late spermatids and oligodendrocytes (Table S6).

Case 29—A male patient with severe multiple morphological anomalies, including microcephaly. *TMEM132C*, *SLC15A4*, and *GLT1D1* from 12q24.32q24.33 were identified as having CNV duplication. *TMEM132C* (transmembrane protein 132C, HGNC:25436) belongs to the protein groups of protein phosphatase 1 regulatory subunits and microRNA protein-coding host genes. Its connection to NDD or its general characteristics have not yet been extensively studied. Like many members of the TMEM family, *TMEM132C* remains poorly characterized. Although the exact functions of *TMEM132C* are still not fully understood, transmembrane proteins (TMEMs) are increasingly recognized for their roles in various nervous system disorders. New findings link TMEMs to diseases such as brain tumors, psychiatric disorders, motor dysfunction, lissencephaly, and neuropathic pain. The involvement of *TMEM132C* in these processes suggests a potential role in neural development and pathophysiology, warranting further functional investigation [46]. Recently, *Tmem132c* was shown to be expressed alongside *Tmem132a* and *Tmem132e* in early neural precursor cells. During mid-pregnancy, distinct localization of *Tmem132c* expression is observed in the oral epithelium, trigeminal ganglia, and structures of the inner ear. Its continued expression in the cerebral cortex and cerebellum during postnatal phases further indicates a role for *TMEM132C* in the maturation of the central nervous system [47]. The gene *SLC15A4* (Solute Carrier Family 15 Member 4, HGNC:23090) is an endolysosomal proton-coupled transporter involved in histidine and dipeptide transport. It plays a key role in immune signaling [48,49,50]. Variants in *SLC15A4* are associated with immune-related diseases, including systemic lupus erythematosus and type 2 diabetes, underscoring its significance in inflammation and innate immunity [51,52]. The gene *GLT1D1* (glycosyltransferase 1 domain-containing 1, HGNC:26483), believed to enable glycosyltransferase activity, has not been extensively studied. However, some findings indicate a role in immune regulation and tumor immune evasion [53]. Additionally, expression data for *GLT1D1* show RNA tissue specificity, with increased expression in the bone marrow, choroid plexus, and liver (Table S6). An expression analysis in key brain regions shows that *GLT1D1* has its lowest expression during the embryonic phase, with levels increasing and stabilizing after birth. In contrast, *SLC15A4* exhibits its highest expression during early embryonic development. The differing expression patterns of *SLC15A4* and *GLT1D1*, which seem to contradict each other, suggest that *SLC15A4* may be involved in early neurological development or immune-related processes, while *GLT1D1* is likely to play a role in the later stages of brain development. Interestingly, all these genes have a low degree and higher betweenness in the smallest custom CMA NDDi and are in a biological network referred to as “connector” or “bottleneck” genes (Figure 2b). They have relatively few direct connections to other genes in the network but play a crucial role in facilitating communication and information flow between various parts of the network. Single-cell expression data could complement their specific tissue functions. While *TMEM132C* is enriched in oligodendrocytes, precursor cells, inhibitory neurons, and microglia, *GLT1D1* is enriched in late spermatids, excitatory neurons, oligodendrocyte precursor cells, dendritic cells, inhibitory neurons, and cone photoreceptor cells, and *SLC15A* is enriched in dendritic cells (Table S6). The CNV duplication 12q24.32–q24.33, which includes *TMEM132C*, *SLC15A4*, and *GLT1D1*, appears to be a strong candidate for further functional studies.

Case 30—A male patient primarily diagnosed with ASD showed no specific morphological features. A CNV duplication was detected in 6q27, encompassing the genes *KIF25* and *FRMD1*. *KIF25* (Kinesin family member 25, HGNC:6390) has recently been identified as a contributor to maintaining centrosome cohesion and supporting proper spindle formation during mitosis, which is essential for accurate chromosome segregation and overall genomic stability [54]. Research has demonstrated that Kinesin family members, like *KIF1C*, play crucial roles in maintaining Golgi organization and facilitating lysosomal transport [55]. This process ultimately results in lysosomal membrane permeabilization and non-apoptotic cell death. Furthermore, some Kinesin family members, like *KIF2C*, *KIF20A*, *KIF18A*, and *KIF15*, have been linked to cancer progression and potentially to resistance against hormone-based therapies [56,57,58]. Within the context of the ASD phenotype, *KIF25* exhibits an expression pattern that suggests a specific role in the central nervous system. RNA expression profiles reveal that *KIF25* is predominantly expressed in neuronal tissues, with transcription frequently occurring in neuronal cell types and expression enhanced in the brain and retina (Table S6). These findings support *KIF25* as a factor in cell division and intracellular organization in neuronal tissues. Next, *FRMD1* (FERM domain-containing 1, HGNC:21240) is believed to regulate Hippo signaling [59]. It exhibits allele-specific DNA methylation in immune cells, suggesting a role in regulating immune genes [60]. Dysregulation of *FRMD1* has been linked to adult T-cell leukemia/lymphoma [61]. Although it is expressed only in certain tissues and is not directly related to the central nervous system (Table S6), a pTriplo of 0.91 indicates a strong candidate for CNV gain pathogenicity.

Case 37—A male patient diagnosed with neurological developmental disorders, including epilepsy and some characteristic facial features. A deletion was identified at 17q21.32, involving the gene *NPEPPS* (aminopeptidase puromycin-sensitive, HGNC:7900). NPEPPS encodes puromycin-sensitive aminopeptidase, an enzyme that degrades enkephalins in the brain [62,63]. Generally, enkephalins play a role in neurotransmission and pain modulation [64]. An interactome analysis has shown that *NPEPPS* has a higher average proximity and a shorter shortest path length, indicating that it is well-connected and can rapidly exchange information with other genes (Figure 2). Expression analysis reveals that NPEPPS is highly and stably expressed in all brain regions from embryonic to end of life (Table S6). Furthermore, a pHaplo score of 0.86 supports the evidence that CNV deletion contributes to the NDD phenotype, making it a strong candidate for the pathogenicity of CNV loss and a compelling candidate for further research.

Summary information of gene functions for novel potential gene–disease associations is presented in Table 5.

## 5. Conclusions

### 5.1. Limitations of the Study

The primary limitation of this study is the small sample size. Additionally, the underlying mechanisms in NDD and ASD can be more complex than simply the deletion or duplication of a single gene. This study focused solely on MANE-selected transcripts to assess their contribution to the cognitive phenotype. Even though the custom array is gene-ordered, non-genic CNVs might indirectly disrupt gene function. Aside from MS-MLPA for Prader–Willi/Angelman syndrome and fragile X syndrome, regions with homozygosity and uniparental disomy were not analyzed. The parental origin of CNVs was not examined. A low mosaicism rate could not be ruled out, and heterochromatic regions (other than GTG bands) were not investigated.

### 5.2. Conclusion and Further Remarks

This study demonstrates that gene-oriented customized CMA enhances the detection of pathogenic and likely pathogenic CNVs in individuals with NDDs and ASD. Beyond improving diagnostic sensitivity, our integrative computational approach enables the prioritization of high-confidence candidate genes with likely putative pathogenic roles in neurodevelopment, offering valuable targets for further functional investigation.

Our case-specific analyses revealed several novel potential gene–disease associations. Duplication involving *PSMG1* and *BRWD1* (Case 10) implicates disruptions in proteasome biogenesis and chromatin remodeling in patients with severe morphological anomalies and microcephaly. *PSMG1* functions as a central network hub, while *BRWD1* is a neuron-enriched regional hub. The deletion of *TPRN* in Case 14 suggests a role in central nervous system myelination, extending its known involvement in auditory function. Case 18 highlights *ZNF92*, a transcription factor expressed in prenatal brain and astrocytes, as a potential contributor to epilepsy and facial dysmorphology. Duplications of *ZNF451* and *FXN* (Case 23) suggest altered transcriptional regulation and DNA repair mechanisms underlying cerebral atrophy and dental anomalies. A CNV involving *TMEM132C*, *SLC15A4*, and *GLT1D1* (Case 29) points to emerging roles in neural development, immune signaling, and network regulation. In Case 30, duplication of *KIF25* and *FRMD1* implicates pathways involved in neuronal cell division and immune regulation. Finally, the deletion of *NPEPPS* in Case 37, a gene with high expression and connectivity in the brain, suggests a pathogenic role in neurotransmission and immune modulation.

Several of the identified candidate genes are involved in immune processes, reinforcing the growing body of evidence potentially linking inflammation to the etiology of NDD. Furthermore, our findings support an expanded view of NDD pathogenesis that includes the involvement of glial cells alongside neuronal dysfunction. Although the study has inherent limitations, our integrative approach—combining genomic, transcriptomic, and network-level data—enhances the robustness of gene prioritization and supports their potential relevance in clinical diagnostics.

## Figures and Tables

**Table 1:** Classification of prioritized custom CMA genes based on centrality metrics in the smallest CMA-NDDi. Up and down arrows in key centrality metrics indicate whether any of the specific parameters are up (up arrow) or down (down arrow).

Classification	Genes	Key Centrality Metrics	Functional Interpretation
High Proximity and Low Shortest Path (Highly Integrated)	*NPEPPS*, *PSMG1*, *DOCK8*, *TUBGCP5*, *FXN*, *ARHGAP11B*, *SULT1A1*, *CBWD1*, *EPHA3*	↑ Proximity, ↓ Shortest Path Length	Highly integrated; likely important in regulating complex cellular processes
Vulnerable/Less Integrated	*KIAA1586*, *ASCL3*, *BTNL3*, *SIRPB1*, *GLT1D1*	↓ Proximity, ↑ Shortest Path Length	Less connected, possibly more susceptible to dysfunction in NDD
Hubs	*DOCK8*, *FXN*, *TUBGCP5*, *PSMG1*, *NPEPPS*, *ARHGAP11B*, *SULT1A1*, *CBWD1*	↑ Degree, ↑ Betweenness	Central “hubs”; major regulators with broad impact if disrupted
Provincial Hubs	*EPHA3*, *BRWD1*, *PDPR*, *CLN8*, *TPRN*, *SHLD2*	↑ Degree, ↓ Betweenness	Locally interactive; may regulate tissue- or compartment-specific functions
Connector/Bottleneck Genes	*SLC15A4*, *TMEM132C*, *GLT1D1*, *SULT1A2* (12q24.32q24.33—CNV gain in one patient)	↓ Degree, ↑ Betweenness	Few connections, but essential for cross-network communication and signal integration

**Table 2:** Summary of extended custom CMA genes in tissue-specific and developmental functional modules.

Category	Key Findings	Example
Gene Integration in Modules	55 out of 56 genes integrated into at least one HumanBase functional module	—
Embryonic Modules (Largest Group)	Most genes found here (46)	*NIPA1*, *SHANK3*, *OR2T10*, *OR2T11*, *OR51A4*
Astrocyte-Specific Modules	Fewer genes (33); despite this, highest number of unique GO terms (25)	*GLUD1* (astrocyte-specific)
Module-Specific Localization	Some genes are enriched in only one module, indicating a highly specialized function	*XKR3* (neuron-M2); *OR2T10/11*, *OR51A4* (embryonic M5)
Prenatal Stage-Specificity	Overlap (40 genes) between fetal and embryonic modules, but also stage-exclusive genes	Embryonic-only: *NIPA1*, *SHANK3* Fetal-only: *GLT1D1*, *KIAA1586*, *TPRN*
Postnatal Integration	Most genes present in multiple modules → broad roles; a few with unique module-specific localization → specialized functions	*CDK11B*, *CLN8*, *DUSP22*, *CBWD1* (cortex); *POTEM*, *ARHGAP11B* (global)
Cell Type Specificity	25 genes common across neuron, glia, and astrocyte modules → broad integration; others show distinct cell-type specificity	Neuron-specific: *CDK11A*, *XKR3*, *SULT1A2 * Glia: *MRGPRX1*, *TFG*, *ARHGAP11B* Astrocyte-only: *GLUD1*
GO Term Enrichment (47 Modules)	235 total GO terms, 123 unique terms	Neurogenesis (GO:0022008) in 8 modules
Most Unique GO Terms by Network	Astrocyte module (25 unique GO terms) > Neuron (10) > Embryo/Fetus/Global (8–9) > Glia, Cortex, CNS, Brain (few unique terms)	—

**Table 3:** Summary of tissue- and cell-specific networks for NDD candidate genes (HumanBase analysis, confidence cutoff > 0.5).

Category	Key Findings	Example Genes
Prenatal Tissue Networks	Few stable interactions; co-functional gene relationships during early development may be transient/context-specific	*SULT1A1*, *SULT1A2*, *SULT1A4*
Postnatal Tissue-Level Networks	Moderate and diffuse interactions across the cerebral cortex, CNS, and global brain	—
Neuronal Cell Network	22 genes with 31 reliable interactions → moderate density	—
Glial Cell Network	Higher density: 26 genes, 77 interactions → underscores glial importance in NDDs	—
Astrocyte Cell Network	Most connected: 36 genes, 181 interactions; supports astrocyte role in synaptic pruning, inflammation, and homeostasis	*GRIA4*, *BOK*, *ZNF92*, *FRMD1*, *KIAA1586*, *CHRFAM7A*
Genes Present in All 3 Cell Networks	9 genes shared across neuron, glia, and astrocyte networks → likely pleiotropic, with roles in circuit development and homeostasis	*PDPR*, *CLN8*, *BRWD1*, *SHLD2*, *GLT1D1*, *SULT1A1*, *ZNF451*, *SULT1A2*, *EPHA3*

**Table 4:** Top-ranking NDD candidate CNVs and clinical findings. In blue are genes that are detected as CNV gain, and in red are genes detected as CNV loss.

Candidate Gene	MANE Transcript	pHaplo	pTriplo	CNV Coordinates/hg38	Total Genes (MANE)	Patient ID (Gender)	Neurodevelopmental Disorders	Comorbidity	Morphological Characteristics
*PSMG1*	NM_003720.4	0.8	0.2	chr21:39175476-39311721	2	10 (f)	epilepsy, undeveloped speech, intellectual disability, immobility, hypertonia, hypotrophy, cerebral atrophy	visual impairment (strabismus), malnutrition	microcephaly, low anterior hairline, facial characteristics (broad face, underdeveloped nasolabial fold), hypertelorism, ear characteristics (protruding underdeveloped ears, long low-set ears), nasal characteristics (short nose, high nasal bridge, short columnella, wide nasal base, concave nasal ridge), smooth philtrum, lip characteristics (absent Cupid’s Bow), high palate, dental disorder, short neck
*BRWD1*	NM_033656.4	0.99	0.89						
*TPRN*	NM_001128228.3	0.46	0.94	chr9:137198240-137204805	2	14 (m)	epilepsy, undeveloped speech, behavioral disorder (hyperactivity), intellectual disability	hydrocephalus, incontinence	brachycephaly, facial characteristics (long face), synophrys
*ZNF92*	NM_152626.4	0.08	0.19	chr7:65179912-65397799	1	18 (m)	epilepsy, intellectual disability	visual impairment (strabismus), scrotal hernia	facial characteristics (asymmetrical face), hypertelorism, ptosis, ear characteristics (protruding ears, underdeveloped antihelix, prominent antitragus, underdeveloped helix), anterior and posterior low hairline, short neck
*FXN*	NM_000144.5	0.2	0.19	chr9:69049545-69251105	2	23 (f)	epilepsy, undeveloped speech, intellectual disability, cerebral atrophy	hirsutism, malnutrition	synophrys, underdeveloped ears, dental disorders, gingival hypertophy, microdontia
*ZNF451*	NM_001031623.3	0.38	0.4	chr6:57048100-57193732	4				
*TMEM132C*	NM_001136103.3	0.99	0.13	chr12:128677379-128877338	3	29 (m)	undeveloped speech, behavioral disorder (hyperactivity, auto agression, stereotypical movements), intellectual disability, hypotonia, hypotrophy	visual impairment, incontinence, cryptorchidism, malnutrition	prominent occiput, mycrocephaly, facial characteristics (cheekbone prominence, sunken cheeks, underdeveloped nasolabial fold), epicanthus, ptosis, nasal characteristics (convex and wide nasal ridge), lip characteristics (thin upper lip), posterior low hairline, alopecia areata
*SLC15A4*	NM_145648.4	0.79	0.06						
*GLT1D1*	NM_144669.3	0.82	0.03						
*KIF25*	NM_030615.4	0.77	0.14	chr6:167938073-168069661	3	30 (m)	behavioral disorder (stereotypical movements, hyperactivity), ASD		
*FRMD1*	NM_024919.6	0.79	0.91						
*NPEPPS*	NM_006310.4	0.86	0.85	chr17:47581475-47597364	1	37 (m)	epilepsy, undeveloped speech, behavioral disorder (sleep disorder, stereotypical movements, hyperactivity), intellectual disability, immobility	incontinence, gastroesophageal reflux disease, hiatal hernia, phimosis, obstipation	facial characteristics (underdeveloped nasolabial folds), horizontal eyebrows

**Table 5:** Summary information of gene functions for novel potential gene–disease associations.

Case	Gene	CNV Type	Function	Brain Expression/Network Role	Disease Associations
10	*PSMG1*	Gain	Proteasome assembly chaperone; forms heterodimer with PSMG2; interacts with PSMA5/PSMA7 [30,31,32]; regulates gene expression via proteasome [33]	Central hub in interactome; broadly expressed in brain with slight decline in fetal → mid stages	Not directly linked to NDDs, but related proteasome genes (PSMB1, PSMC3) linked to autism, delays, microcephaly [34,35]
	*BRWD1*		Transcription regulator; chromatin remodeling [36]; regulates morphology and cytoskeleton (BRWD1_HUMAN, Q9NSI6)	Provincial hub; expressed in neuronal, glial, astrocyte networks; synaptic expression profile	Increased brain expression; pTriplo = 0.89; no CNV in DGV
14	*TPRN*	Loss	Sensory epithelial protein; linked to non-syndromic deafness [37]; involved in CNS myelination [38] and axon sheathing [39]	Cell-type-specific to oligodendrocytes; fetal network; provincial hub	No CNV in DGV; functionally linked to oligodendrocyte-specific expression
18	*ZNF92*	Gain	DNA-binding transcription factor; Pol II-specific; cis-regulatory binding (Alliance of Genome Resources, Jun 2025); nuclear localization (Mar 2025)	Astrocyte-specific expression; high prenatal expression sharply declines postnatally	No CNV in DGV; candidate for astrocyte-linked transcriptional regulation
23	*ZNF451*	Gain	SUMO ligase; transcriptional co-repressor; involved in DNA repair via TDP2/TOP2 complex resolution [40,41]; transcriptional regulation via SUMOylation [42]; affects cancer therapy resistance [43,44,45]	Dynamically expressed across brain; enriched in late spermatids, oligodendrocytes	Linked to cancer progression; potential NDD relevance via expression and repair pathways
29	*TMEM132C*	Gain	Poorly characterized TMEM; linked to brain tumors, psychiatric disorders, lissencephaly, etc. [46]; expressed in early neural precursors [47]	Expressed in oligodendrocytes, precursors, inhibitory neurons, microglia; connector/bottleneck gene	Candidate for CNS development role; warrants further study
	*SLC15A4*		Endolysosomal proton-coupled transporter; histidine/dipeptide transport [48,49,50]; immune signaling [51]; linked to autoimmune disease [52]	Expressed in dendritic cells; high embryonic expression; connector/bottleneck gene	Linked to innate immunity
	*GLT1D1*		Putative glycosyltransferase; may aid in immune regulation/tumor evasion [53]	Enriched in neurons, dendritic cells, cone photoreceptors; low prenatal, increasing postnatally; connector gene	Expressed in bone marrow, choroid plexus, liver
30	*KIF25*	Gain	Kinesin motor protein; centrosome cohesion; mitosis, lysosomal transport [54,55]; involved in non-apoptotic cell death; linked to cancer [56,57,58]	Neuronal-specific expression; enriched in brain and retina	Role in cell division and organization; candidate for ASD based on expression
	*FRMD1*		Regulates Hippo signaling [59]; allele-specific methylation in immune cells [60]; linked to T-cell leukemia [61]	Not brain-specific	Immune regulation role; pTriplo = 0.91
37	*NPEPPS*	Loss	Encodes aminopeptidase; degrades enkephalins in brain [62,63]; involved in neurotransmission/pain modulation [64]	Highly expressed in brain throughout lifespan; well-connected in interactome	Strong CNV deletion candidate (pHaplo = 0.86); implicated in NDD with epilepsy

## Data Availability

The original contributions presented in this study are included in the article/Supplementary Material. Further inquiries can be directed to the corresponding author(s).

## Supplementary Materials

The following supporting information can be downloaded at: https://www.mdpi.com/article/10.3390/genes16080868/s1, Supplementary Table S1. A complete list of all detected CNV regions. Supplementary Table S2. A custom CMA gene list. Supplementary Table S3. A complete list of all lncRNAs. Supplementary Table S4. Protein-protein interactions for the 61 MANE custom CMA gene list based on their first neighbors. Supplementary Table S5. The integrative “NDD gene panel”. Supplementary Table S6. Gene network with 824 nodes and 1221 edges (sub-interactome a). Supplementary Table S7. Gene network with 207 nodes and 278 edges (sub-interactome b). Supplementary Table S8. CMA-NDD interactome with 1004 nodes and 1498 edges. Supplemental Table S9. Complete list of genes presented in the smallest custom CMA NDD interactome. Supplemental Table S10. The prioritized gen list, denoting a list of custom CMA NDD genes present in the smallest custom CMA NDD interactome. Supplementary Table S11. List of pathogenic and likely pathogenic CNVs detected by 180K and custom 60K CMA. Genomic coordinates for 180K are in black and red for 60K. Supplementary Table S12. List of pathogenic and likely pathogenic CNVs detected by custom 60K CMA. Supplementary Table S13. Interactome analysis summary. Supplementary Table S14. Genes from the CMA list found in the “NDD gene panel”. * Genes present in the smallest custom CMA NDDi from both the custom CMA gene list and “NDD gen panel”. Supplementary Table S15. Tissue-specific network-based functional modules for the expanded CMA gene list from The HumanBase. Supplementary Table S16. The top five GO terms for nine tissue networks from The HumanBase. Supplementary Table S17. Unique and shared GO terms for tissue-specific, network-based functional modules. Supplementary Table S18. The MGI database searches for matching mammalian phenotypes. Supplementary Table S19. The Database of Genomic Variants (Gold Standard) search. Supplementary Table S20. Overview of the CNVs with candidate genes found in the patients arranged by chromosomal location. Supplementary Table S21. Expression profile for candidate genes. Figure S1. Embryonic and fetal tissue-specific gene networks for prioritized custom CMA genes. Figure S2. Postnatal brain tissue-specific gene networks for prioritized custom CMA genes. Figure S3. Cell-type-specific gene networks for prioritized custom CMA genes in neurons, glia, and astrocytes. Figure S4. Structural variation profiles of prioritized candidate genes from the Database of Genomic Variants (DGV).

## Abbreviations

The following abbreviations are used in this manuscript:

CMA	Chromosomal Microarray
NDD	Neurodevelopmental Disorders
CNV	Copy Number Variation
ASD	Autism Spectrum Disorder
GO	Gene Ontology
MGI	Mouse Genome Informatics
pLI	Probability of being Loss-of-function-Intolerant
LOEUF	Loss-of-function Observed/Expected Upper bound Fraction
sHet	Selection coefficient against heterozygous loss-of-function variants
pHaplo	Probability of haploinsufficiency
pTriplo	Probability of triplosensitivity
HIPPIE	Human Integrated Protein–Protein Interaction rEference
NDEx	Network Data Exchange
SFARI	Simons Foundation Autism Research Initiative
OMIM	Online Mendelian Inheritance in Man
GTG	Giemsa (G-banding technique)
MLPA	Multiplex Ligation-dependent Probe Amplification
DGV	Database of Genomic Variants
SNV	Single Nucleotide Variant
indel	Insertion-deletion (variant)
CGH	Comparative Genomic Hybridization
MANE	Matched Annotation from NCBI and EMBL-EBI
UCSC	University of California, Santa Cruz (Genome Browser)
GRCh38	Genome Reference Consortium Human Build 38
RNA	Ribonucleic Acid
TF	Transcription Factor
lncRNA	Long Non-Coding RNA
STRING	Search Tool for the Retrieval of Interacting Genes/Proteins
MCL	Markov Cluster Algorithm
