# Supplementary material for: Customized Chromosomal Microarrays for Neurodevelopmental Disorders
Source: Genes (Basel). 2025 Jul 24;16(8):868. doi: 10.3390/genes16080868 (PMC12385676; doi:10.3390/genes16080868)

Figure S1. Embryonic and fetal tissue-specific gene networks for prioritized custom CMA genes. Tissue-specific functional modules were constructed using the HumanBase database to assess prenatal expression patterns and gene network integration. The networks represent embryonic and fetal developmental stages and include CMA-prioritized genes mapped to their respective functional modules. A total of 40 genes were shared between embryonic and fetal networks, reflecting conserved functions across early developmental windows. Several genes showed stage-specific expression: Embryo-specific genes: NIPA1, SHANK3, NUTM2A, OR2T10, OR2T11, and OR51A4. Fetus-specific genes: GLT1D1, KIAA1586, and TPRN.

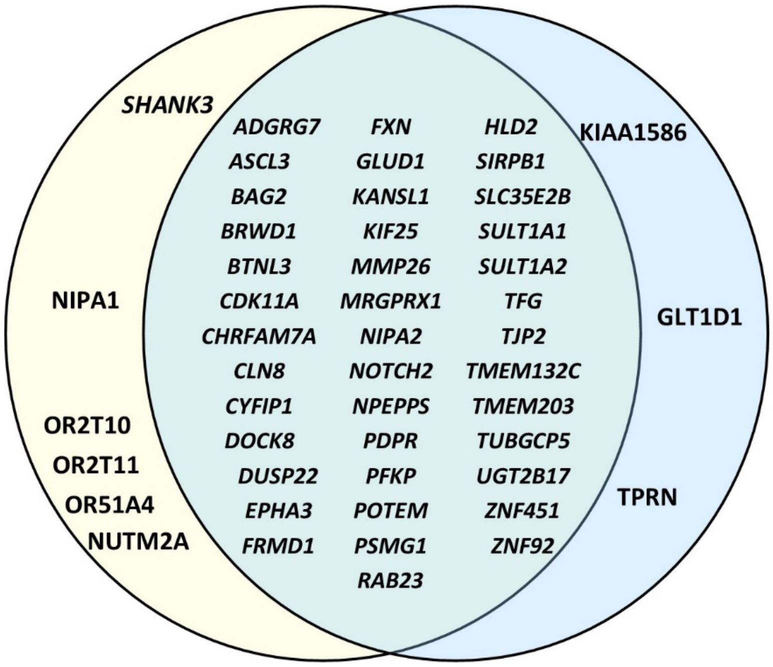

Figure S2. Postnatal brain tissue-specific gene networks for prioritized custom CMA genes. Tissue-specific functional modules were constructed using HumanBase for global brain, central nervous system (CNS), brain, and cerebral cortex to investigate postnatal gene function. Most custom CMA genes were integrated into multiple tissue-specific modules, suggesting broad postnatal relevance. A few genes showed tissue-specific enrichment, appearing in only one module: CDK11B, CLN8, DUSP22, and CBWD1 were specific to the cerebral cortex network. POTEM and ARHGAP11B were unique to the global brain network.

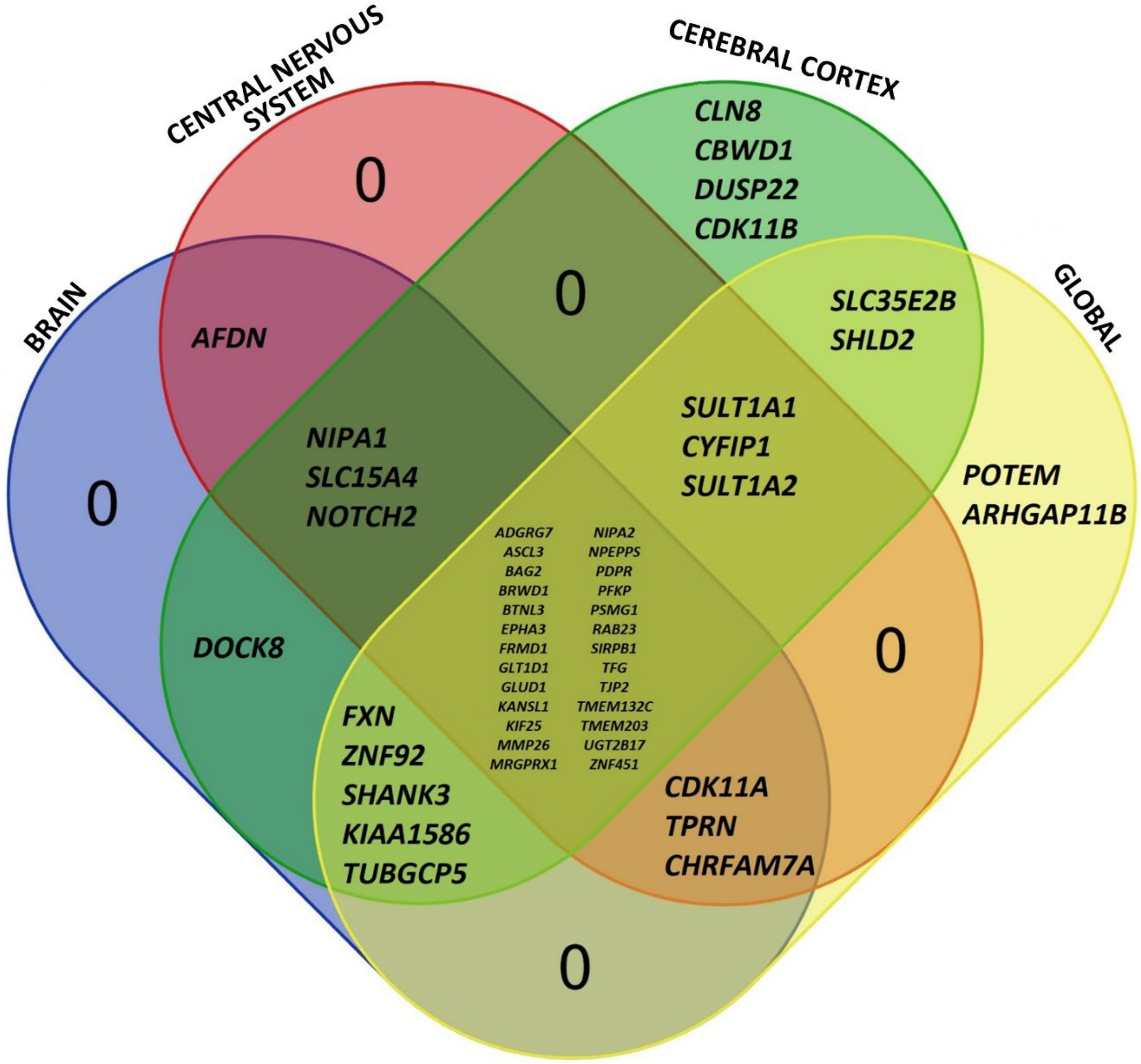

Figure S3. Cell type–specific gene networks for prioritized custom CMA genes in neurons, glia, and astrocytes. To assess cellular-level functional relevance, HumanBase-derived gene networks were generated for neurons, glial cells, and astrocytes. A majority of genes (25) were present in all three networks, indicating broad involvement in diverse CNS cell types. Distinct cell type–specific genes were also observed: Neuron-specific: CDK11A, XKR3, ADGRG7, ZNF451, SULT1A2 Glia-specific: MRGPRX1, CDK11B, ARHGAP11B, TFG Astrocyte-specific: GLUD1.

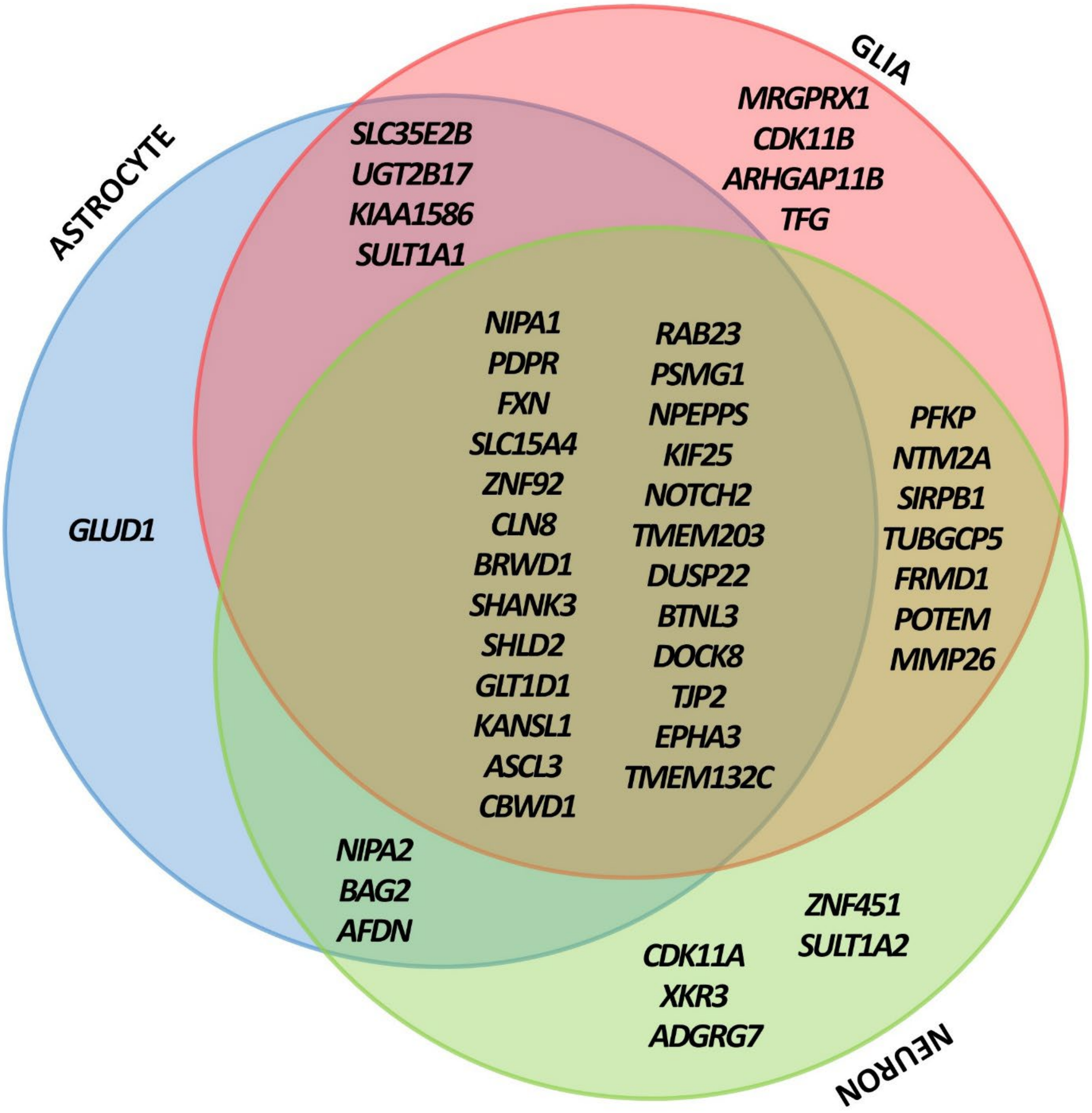

Figure S4. Structural variation profiles of prioritized candidate genes from the Database of Genomic Variants (DGV). This figure illustrates the distribution and internal ranking of structural variants (SVs) affecting prioritized custom CMA genes, using data from the DGV Gold Standard. Most candidate genes showed low-frequency or minor structural variants, suggesting potential pathogenic relevance when altered. Several genes (*ZNF92*, *CLN8*, *TPRN*, *PSMG1*, *BRWD1*) had no reported CNVs in the DGV.

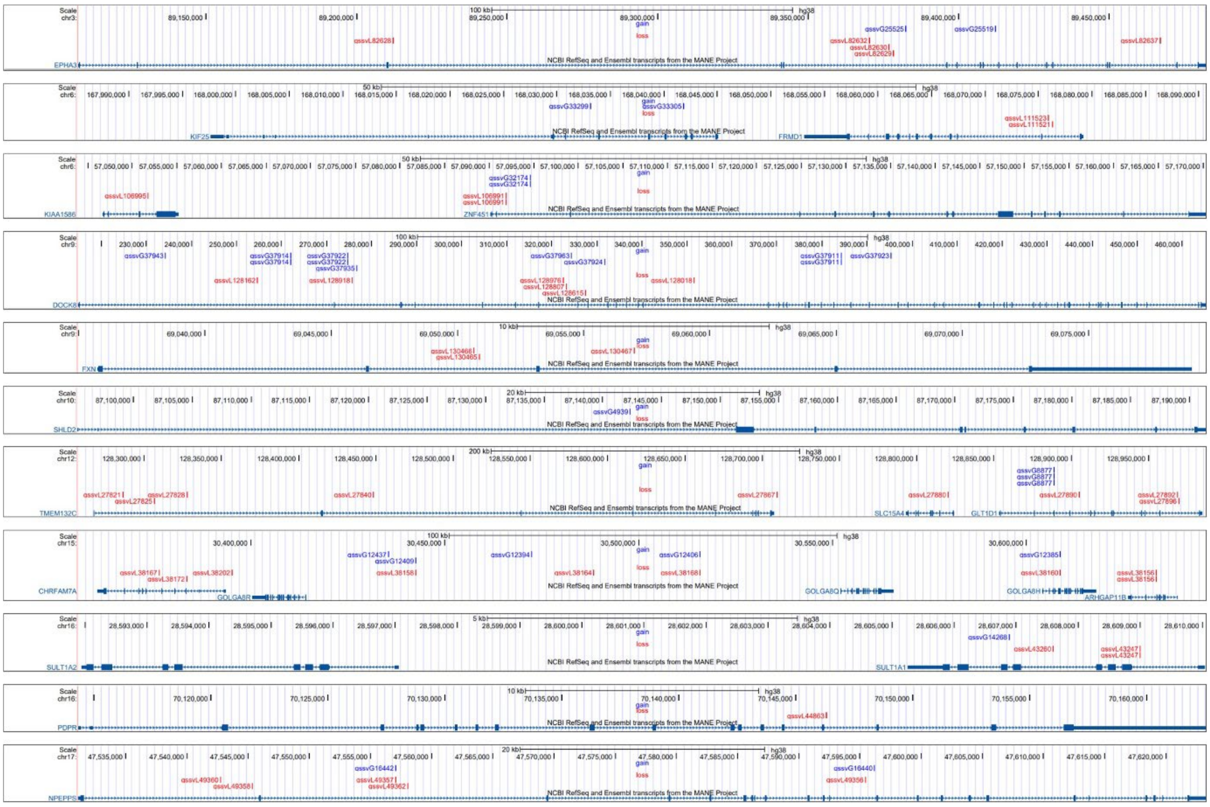

Supplement: Supplementary file 1 [file genes-16-00868-s001.zip › Figure S1-S4.pdf]
